# Serum Biochemistry and Histopathological Profiles of Normoglycemic and Streptozotozin-Induced Hyperglycemic Rats Treated with Mace Water Extract from *Myristica fragrans* Houtt

**DOI:** 10.3390/nu18162686

**Published:** 2026-08-18

**Authors:** Hasbullah, Fitriya Nur Annisa Dewi, Didah Nur Faridah, Dias Indrasti, Silvia Arin Prabandari, Nuri Andarwulan, Dominika Średnicka-Tober

**Affiliations:** 1 Faculty of Agriculture, Khairun University, Ternate 97719, North Maluku, Indonesia; 2 School of Veterinary Medicine and Biomedical Science, IPB University, Bogor 16680, West Java, Indonesia; fitriyanur@apps.ipb.ac.id; 3 Primate Research Center, IPB University, Bogor 16680, West Java, Indonesia; viviarin@gmail.com; 4 Department of Food Science and Technology, Faculty of Agricultural Technology, IPB University, IPB Dramaga Campus, Bogor 16680, West Java, Indonesia; didah_nf@apps.ipb.ac.id (D.N.F.); d_indrasti@apps.ipb.ac.id (D.I.); 5 Southeast Asian Food and Agricultural Science and Technology (SEAFAST) Center, IPB University, IPB Dramaga Campus, Bogor 16680, West Java, Indonesia; 6 Department of Functional and Organic Food, Institute of Human Nutrition Sciences, Warsaw University of Life Sciences, Nowoursynowska 159c, 02-776 Warsaw, Poland

**Keywords:** aril, diabetes mellitus, functional foods, histopathology, hyperglycemic, myristica, novel foods, nutmeg, streptozotocin, Sprague Dawley

## Abstract

**Background/Objectives:** This in vivo study was conducted to investigate the effects of mace water extract from *Myristica fragrans* Houtt (ME) on serum biochemical and histopathological profiles of normoglycemic and streptozotozin-induced hyperglycemic rats. **Methods:** Biochemical parameters (cholesterol, HDL, LDL, triglycerides, creatinine, blood urea nitrogen, AST, ALT, and albumin) from all groups of experimental rats were analyzed. Histopathological analysis was also performed on the kidney, liver, and pancreatic tissues. **Results:** The results showed that administration of ME (1.84 mg total phenolics from ME/kg BW) was able to prevent serum biochemical changes that are indicators of metabolic disorders and organ tissue damage due to hyperglycemia. ME significantly reduced LDL (31.85%), creatinine (13.78%), AST (58.97%), and ALT (60.04%) levels in hyperglycemic rats. ME also showed promising performance in protecting the kidney and liver from damage due to hyperglycemia. **Conclusions:** The findings in this study emphasize the potential of ME to be used as a therapeutic agent and functional ingredient.

## 1. Introduction

Diabetes mellitus is a chronic metabolic disease that poses a major challenge to the global health system. This disease is characterized by hyperglycemia caused by impaired insulin secretion, insulin resistance, or a combination of both [1]. Chronic hyperglycemia not only affects glucose metabolism but also causes lipid and protein metabolism disorders that contribute to various systemic complications. In addition to the pancreas, vital organs such as the liver, kidneys, and cardiovascular system are highly susceptible to damage from long-term hyperglycemia [2]. According to the latest global epidemiology report from the International Diabetes Federation, the number of adults with diabetes worldwide reached approximately 589 million in 2024 and is expected to increase to 853 million by 2050 [3]. This increasing prevalence makes diabetes one of the leading causes of global morbidity and mortality.

Chronic hyperglycemia can trigger various pathophysiological mechanisms that contribute to tissue damage, including oxidative stress, the formation of advanced glycation end products (AGEs), activation of the polyol pathway, and mitochondrial dysfunction [4,5,6]. These mechanisms lead to increased production of reactive oxygen species (ROS), disrupting cellular redox balance. The accumulation of ROS ultimately causes damage to biomolecules such as lipids, proteins, and DNA, which play a key role in the development of diabetic complications [7,8]. This condition also affects various serum biochemical parameters often used as clinical indicators of metabolic status and organ function.

Diabetic patients generally show increased levels of total cholesterol, triglycerides, and low-density lipoprotein (LDL) and decreased levels of high-density lipoprotein (HDL) [9,10,11,12]. This dyslipidemia contributes to an increased risk of atherosclerosis and cardiovascular disease, which are the leading causes of death in diabetics [13]. Furthermore, diabetes is also associated with changes in kidney function parameters such as azotemia or increased creatinine and/or blood urea nitrogen (BUN) levels, reflecting impaired glomerular filtration due to diabetic nephropathy [14,15]. At the hepatic level, increased activity of the transaminase enzymes, aspartate aminotransferase (AST) and alanine aminotransferase (ALT), is frequently reported in individuals with diabetes as an indicator of hepatocellular damage related to impaired lipid metabolism and oxidative stress [16,17,18]. Serum albumin is also an important indicator of nutritional and metabolic status and is frequently altered in chronic degenerative diseases, including diabetes [19].

In biomedical research, experimental animal models are essential tools for understanding disease pathogenesis and evaluating the effectiveness of new therapies. One of the most widely used models in diabetes research is the streptozotocin (STZ)-induced rat model. Streptozotocin is a nitrosourea compound with a high affinity for pancreatic β-cells due to its transport by the GLUT2 glucose transporter [20,21]. This compound causes β-cell damage through DNA alkylation, activation of poly-ADP-ribose polymerase, and increased free radical production, leading to cell apoptosis. As a result of pancreatic β-cell damage, insulin production decreases significantly, resulting in hyperglycemia [22]. The STZ-induced rat model is widely used in diabetes research because it mimics the metabolic and pathological changes that occur in human diabetes. In individuals with naturally occurring type 2 diabetes, the pancreas initially overproduces insulin to compensate for the insulin resistance, and, over time, this overwork results in damage and a decrease in the number of β cells, which further play an important role in regulating glucose homeostasis [23,24].

In addition to the pancreas, the kidney is one of the organs most susceptible to damage caused by chronic effects of hyperglycemia, wherein the animal models often show glomerular hypertrophy, thickening of the glomerular basement membrane, expansion of the mesangial matrix, and tubular degeneration [25]. These changes contribute to the development of diabetic nephropathy, which is the main cause of chronic kidney failure in diabetics [26,27]. Diabetes is also often associated with the development of metabolic dysfunction-associated steatotic liver disease (MASLD), which is characterized by lipid accumulation in hepatocytes, inflammation, and liver cell damage [28]. At the histopathological level, this condition can be observed through changes such as hepatic steatosis, hepatocyte degeneration, and inflammatory cell infiltration [29].

In recent years, there has been increasing interest in the use of natural products for the management of diabetes mellitus. Medicinal plants are known to contain various bioactive compounds such as flavonoids, polyphenols, alkaloids, and terpenoids that exhibit antioxidant, anti-inflammatory, and antihyperglycemic activities [30,31,32,33,34,35,36,37,38,39,40]. These compounds have been reported to help reduce oxidative stress, increase insulin sensitivity, and improve glucose and lipid metabolism [41,42,43]. Therefore, exploring medicinal plants as sources of bioactive compounds for diabetes therapy is a rapidly growing field of research [44].

One tropical plant with pharmacological potential is nutmeg (*Myristica fragrans* Houtt) [45], which has long been used as a spice and traditional medicine in various tropical countries [46,47]. Nutmeg is known to contain various bioactive compounds, such as lignans, phenylpropanoids, flavonoids, and essential oils, with diverse biological activities, including antioxidant, anti-inflammatory, and antihyperglycemic [48,49,50]. Several studies have reported that *Myristica fragrans* extract has antihyperglycemic activity in animal models of diabetes [51]. Bioactive compounds in nutmeg are known to inhibit the enzymes α-glucosidase and α-amylase, which play a role in carbohydrate digestion, thereby reducing the increase in postprandial blood sugar [52]. In addition, the antioxidant activity of phenolic compounds in nutmeg also plays a role in protecting pancreatic β cells from oxidative stress-induced damage [53,54]. Other studies have shown that nutmeg extract can improve lipid profiles and reduce tissue damage in animal models of diabetes.

One part of the nutmeg plant that has attracted attention in pharmacological research is mace, the aril that covers the nutmeg seed. Mace contains various bioactive compounds that can potentially serve as antidiabetics and antioxidants [36,37,38,55,56]. To date, there is limited data available on the effect of mace extract on serum biochemical profiles related to lipid metabolism, liver function, and kidney function in animal models of diabetes. Furthermore, in the available studies on mace, methanol or ethanol extraction were used. While the applicability of organic solvents (including methanol) in the production of food is generally limited due to safety considerations, studies using water for extracting mace are not yet available. The use of water extraction offers several advantages, including safety, ease of application, and potential use in the development of functional food or nutraceutical products. Therefore, research evaluating the effects of mace water extracts from *Myristica fragrans* (ME) on metabolic and histopathological changes in a rat model of hyperglycemia is novel and highly relevant.

Based on this background, this study aims to evaluate the serum biochemical and histopathological profiles in normoglycemic and STZ-induced hyperglycemic rats after administration of ME. Biochemical parameters analyzed included triglycerides, total cholesterol, high-density lipoprotein (HDL) cholesterol, low-density lipoprotein (LDL) cholesterol, creatinine, BUN, transaminases (AST and ALT), and albumin, whereas histopathological analysis was performed on pancreatic, kidney, and liver tissues.

The study is a continuation of research on the in vitro antioxidant and antidiabetic activity [36], and the in vivo antihyperglycemic potential [38] of *Myristica fragrans* Houtt mace water extract. While our previous publications focused on (a) the mace extract’s phenolic content, antioxidant activity and α-amylase inhibitory activity in vitro [36], as well as (b) measurements of fasting blood glucose, oral starch and glucose tolerance tests, glycated hemoglobin (HbA1c), water consumption, body weight, and relative weight of organs of experimental animals in the in vivo experiment [38], the results of the current study were expected to provide further, more comprehensive scientific information regarding the potential of ME as a natural agent for preventing or alleviating metabolic disorders and tissue damage associated with diabetes mellitus.

## 2. Materials and Methods

### 2.1. Sampling and Extraction

The mace of *Myristica fragrans* Houtt was collected from nutmeg trees cultivated by local farmers in Dorpedu Village, North Maluku Province, Indonesia. The botanical identity of the plant material was verified by Herbarium Bogoriense, Botany Research Center–Indonesian Institute of Sciences. The following are specimen numbers from plants identified by the LIPI (Indonesian Institute of Sciences) Biology Research Center: B-181/IV/DI.01/1/2021. The dried mace was pulverized and subsequently passed through a 30-mesh sieve to obtain a uniform powder. Immediately after grinding, the mace powder was tightly sealed in plastic bags containing silica gel and stored under frozen conditions for no longer than two weeks prior to use.

The extraction procedure was performed according to the method described by Hasbullah et al. [36]. Briefly, 25 g of mace powder was mixed with 250 mL of distilled water at a 1:10 (*w*/*v*) ratio and subjected to sonication for 30 min at 30 °C. The suspension was then passed through V60 filter paper (first filtration), and the remaining residue was extracted again using the same procedure. The filtrate obtained from the first filtration was subsequently passed through Whatman No. 1 filter paper (second filtration). The resulting filtrate was concentrated using a rotary evaporator and subsequently freeze-dried (Labconco 7052040, SN050841958A, Kansas City, MO, USA) until a dry mace extract (ME) was obtained.

### 2.2. Experimental Animals

Healthy male Sprague Dawley rats, aged 5 weeks, were obtained from PT. BMTI, Bogor, Indonesia, and housed under controlled conditions with a 12:12 h light–dark cycle at 25 ± 2 °C. Feed and distilled water were provided ad libitum. The animals received a standard rodent diet (PT. INDO FEED) containing 20% protein, 4% fat, 4% fiber, 4% ash, 0.8% phosphorus, 1.25% arginine, 0.9% alanine, and 12% water, with an estimated energy content of 2750 kcal/kg. Clinical parameters assessed included body weight, daily water intake, and organ weight. The water consumption pattern, as well as the effects of the experimental treatments on body and organ weights, have been reported previously by Hasbullah et al. [38]. All animal housing and experimental procedures were conducted in accordance with established ethical and animal welfare guidelines and were approved by the Animal Ethics Committee of the School of Veterinary Medicine and Biomedical Sciences, IPB University (Approval No. 065/KEH/SKE/VI/2023).

### 2.3. Experimental Design

The sample size was determined using the resource equation method [57], which provides a recommended range for the minimum and maximum number of animals required for experimental studies. Based on this approach, the estimated sample size ranged from 3 to 5 animals per group. Considering the 28-day experimental period and the possibility of disease or mortality among the animals, the maximum number (*n* = 5) was selected. For the intervention study, rats with a mean body weight of 271.84 ± 9.84 g were randomly allocated to five experimental groups, with five animals in each group (*n* = 5). The groups were as follows: (1) normal rats receiving distilled water without further intervention (normal control); (2) hyperglycemic rats receiving distilled water without treatment (negative control); (3) hyperglycemic rats treated with acarbose at 10 mg/kg BW (positive control); (4) hyperglycemic rats administered mace extract (ME) at 75 mg/kg BW; and (5) normal rats administered ME at 75 mg/kg BW. The number of hyperglycemic group animals that survived to the end of the study period was 3 in each group, still meeting the minimum sample size requirement (*n* = 3) according to the applied sample size determination method.

For administration, the dried mace extract was reconstituted in distilled water. The dose of 75 mg ME/kg BW was selected based on previously published findings [38], where this dose represented the lowest tested concentration that resulted in a lower area under the curve (AUC) than that observed in the negative control during the oral starch tolerance test. In addition, previous analysis [36] showed that the mace aqueous extract used in the present study contained 24.53 ± 2.86 mg GAE/g ME. Accordingly, administration of 75 mg ME/kg BW corresponded to an estimated intake of 1.84 mg total phenolics/kg BW. This dose was substantially below the previously reported safety limits [58,59].

Hyperglycemia was induced in rats by a single intraperitoneal administration of streptozotocin (50 mg/kg BW), prepared in 0.1 M citrate buffer (pH 4.5), following an overnight fasting period at 9 weeks of age. Seven days after streptozotocin administration, fasting blood glucose concentrations were measured. Rats with fasting blood glucose levels exceeding 250 mg/dL were classified as hyperglycemic and subsequently included in the next stage of the experiment.

The test materials were administered orally once daily for 28 consecutive days using a gastric gavage. At the end of the experimental period, all rats were fasted overnight and subsequently humanely euthanized by exsanguination under deep anesthesia with ketamine and xylazine. Blood samples were collected via cardiac puncture, followed by serum separation by centrifugation at 3000 rpm for 20 min. The resulting serum samples were immediately stored under frozen conditions until further analysis. Samples of the pancreas, liver, and kidneys were also collected and fixed in 10% neutral buffered formalin for subsequent histopathological evaluation.

### 2.4. Blood Biochemical Parameter Tests

Serum samples were used to evaluate triglycerides, total cholesterol, HDL cholesterol, LDL cholesterol, BUN, creatinine, albumin, ALT, and AST levels using Mindray BA-88A Semi-Auto Chemistry Analyzer and Photometer and reagent kits from Glory Diagnostics (Barcelona, Spain) and BioMaxima (Lublin, Poland). Results of other measurements taken within the study (i.e., fasting blood glucose, oral starch and glucose tolerance tests, glycated hemoglobin (HbA1c)) were previously reported by Hasbullah et al. [38].

### 2.5. Histopathological Analyses

Pancreatic, liver, and kidney tissues fixed in formalin were processed and embedded in paraffin. Tissue sections, 5 μm in thickness, were then prepared from the paraffin blocks and stained with hematoxylin and eosin (H&E) for histopathological evaluation. The hematoxylin and eosin (H&E) staining was used in this study as the standard method for structural tissue examination, facilitating assessment of overall tissue architecture and islet morphology, although not allowing for β-cell differentiation.

Tissue slides were examined using a light microscope (Nikon Microscope ECLIPSE 80i, Nikon Corporation, Mito City, Japan) and photomicrographs (Nikon Digital Sight DS-Fi1, Nikon Corporation, Otawara City, Japan) were taken at 200× magnification [60]. For each pancreatic tissue, the area of Langerhans islands was measured in 10 low-powered field images using the ImageJ software (version 1.54p). Descriptive tissue morphology evaluation of the kidney and liver was performed by a certified veterinary pathologist. All histopathological analyses were performed blinded to the experimental subjects and grouping.

### 2.6. Statistical Analysis

Statistical analysis was performed with SPSS version 26. Normality of the data was evaluated using the Shapiro–Wilk test, and statistical analysis was performed by one-way ANOVA, followed by Duncan’s test for post-hoc pairwise comparisons. Results are presented as mean values with standard deviation (SD). Statistical analysis with the result of *p* < 0.05 was considered significant.

## 3. Results

### 3.1. Lipid Profile (Cholesterol, Triglycerides, HDL, and LDL)

**Figure 1:**
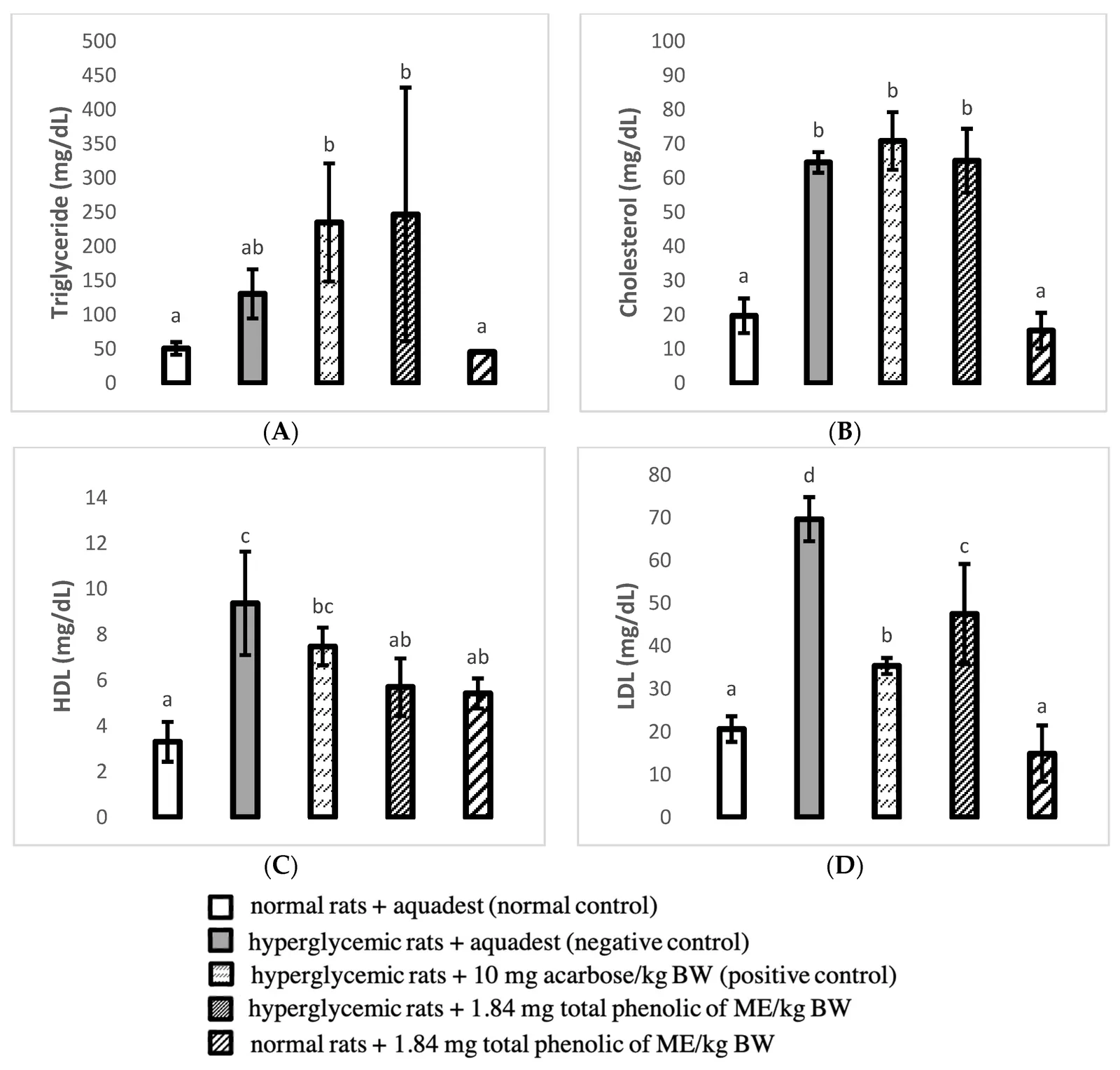
Lipid profile of experimental rats: (**A**) Triglyceride; (**B**) Cholesterol; (**C**) HDL; (**D**) LDL. Different lowercase letters above bars indicate statistically significant differences (*p* < 0.05).

Induction of hyperglycemia with STZ significantly altered the lipid profile and triggered dyslipidemia in experimental rats (*p* < 0.05) (Figure 1A–D). This was characterized by a spike in levels of total cholesterol (Figure 1B), HDL (Figure 1C) and LDL (Figure 1D) cholesterols compared to normal controls.

Administration of ME did not significantly reduce total cholesterol or triglycerides when compared to hyperglycemic rats given aquadest as a placebo or negative control (Figure 1A,B). However, the extract showed a prominent effect in modulating lipoprotein fractions, particularly in lowering HDL to 5.70 ± 1.25 mg/dL and LDL to 47.50 ± 11.72 mg/dL levels in the hyperglycemic rat group. For LDL, this effect was less prominent when compared to the effect of acarbose. Notably, however, the large standard deviation indicates a wide range of individual responses of rats to the treatment.

### 3.2. Markers of Kidney Function (Creatinine and BUN)

**Figure 2:**
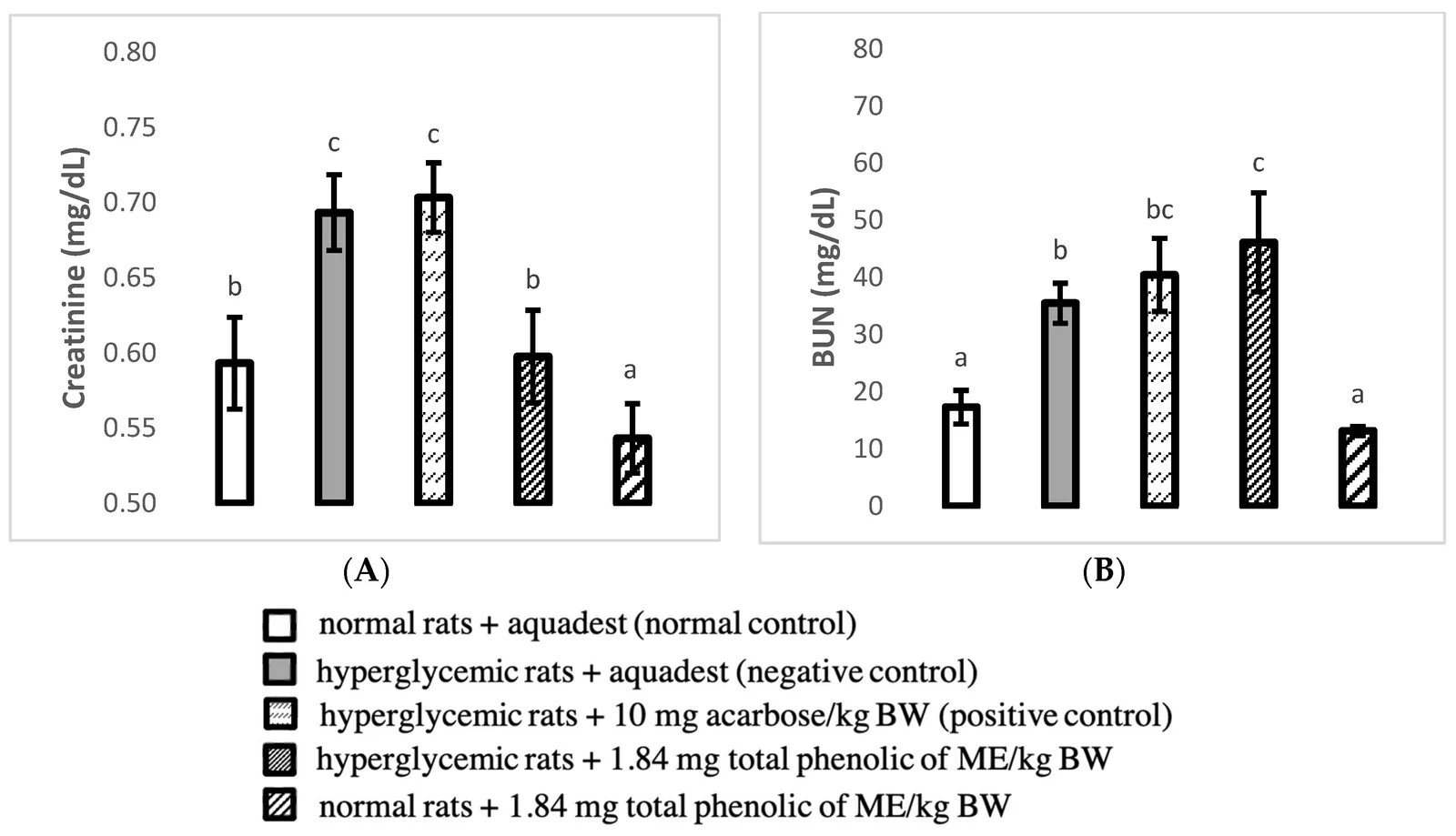
Serum biochemical parameters of kidney function in experimental rats: (**A**) Creatinine; (**B**) Blood Urea Nitrogen (BUN). Different lowercase letters above bars indicate statistically significant differences (*p* < 0.05).

The negative control group showed a significant spike (*p* < 0.05) in blood urea nitrogen (BUN) (35.52 ± 3.51 mg/dL) and creatinine (0.69 ± 0.03 mg/dL), compared to the normal rats (Figure 2A,B), which confirmed azotemia occurring in the hyperglycemic rats that may be due to nephrotoxicity resulting from STZ induction. Treatment with ME showed a significantly lower creatinine than the negative control and the positive control (acarbose) (*p* < 0.05), notably comparable to the creatinine level of the normal group. The level of BUN in hyperglycemic rats, however, was significantly higher with extract treatment when compared to aquadest (*p* < 0.05), but it did not differ from the acarbose effect.

### 3.3. Liver Function Enzymes (AST, ALT) and Albumin

**Figure 3:**
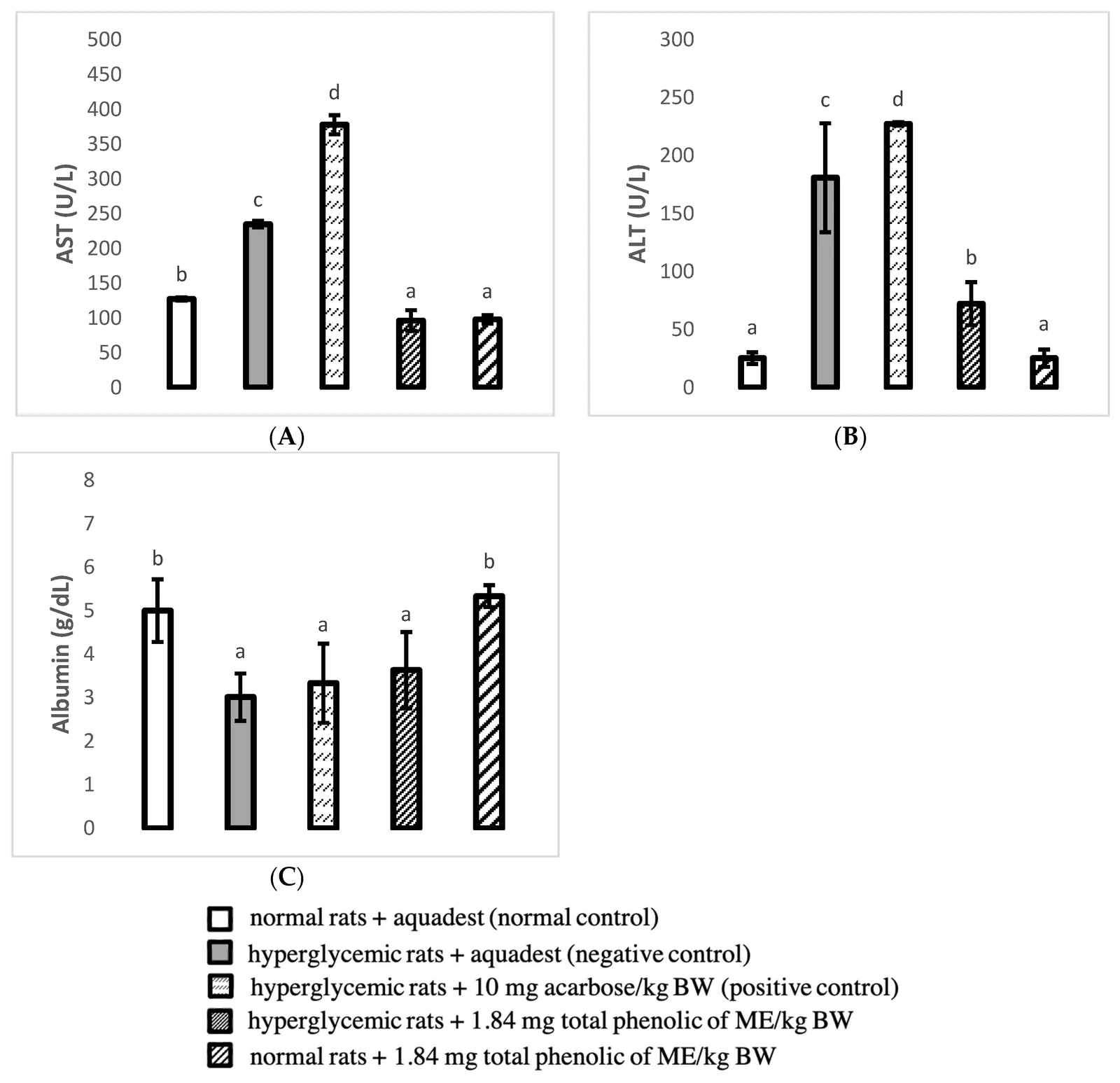
Serum biochemical parameters of experimental animals as markers of hepatotoxicity: (**A**) AST; (**B**) ALT; and (**C**) Albumin. Different lowercase letters above bars indicate statistically significant differences (*p* < 0.05).

Significantly higher levels of AST (234.88 ± 4.71 U/L) and ALT (181.00 ± 47.00 U/L) were observed (*p* < 0.05) in the negative control group compared to the normal control (127.39 ± 2.45 U/L and 25.33 ± 5.03 U/L, respectively), which indicated hepatocyte leakage in hyperglycemic rats (Figure 3A,B). Interestingly, hyperglycemic rats treated with ME showed significantly lower ALT (72.33 ± 18.58 U/L) and AST (96.37 ± 14.80 U/L) levels than both negative and positive controls. On the other hand, albumin levels in all groups of hyperglycemic rats were higher compared to the normal groups (*p* < 0.05) (Figure 3C).

### 3.4. Histopathological Profile

**Figure 4:**
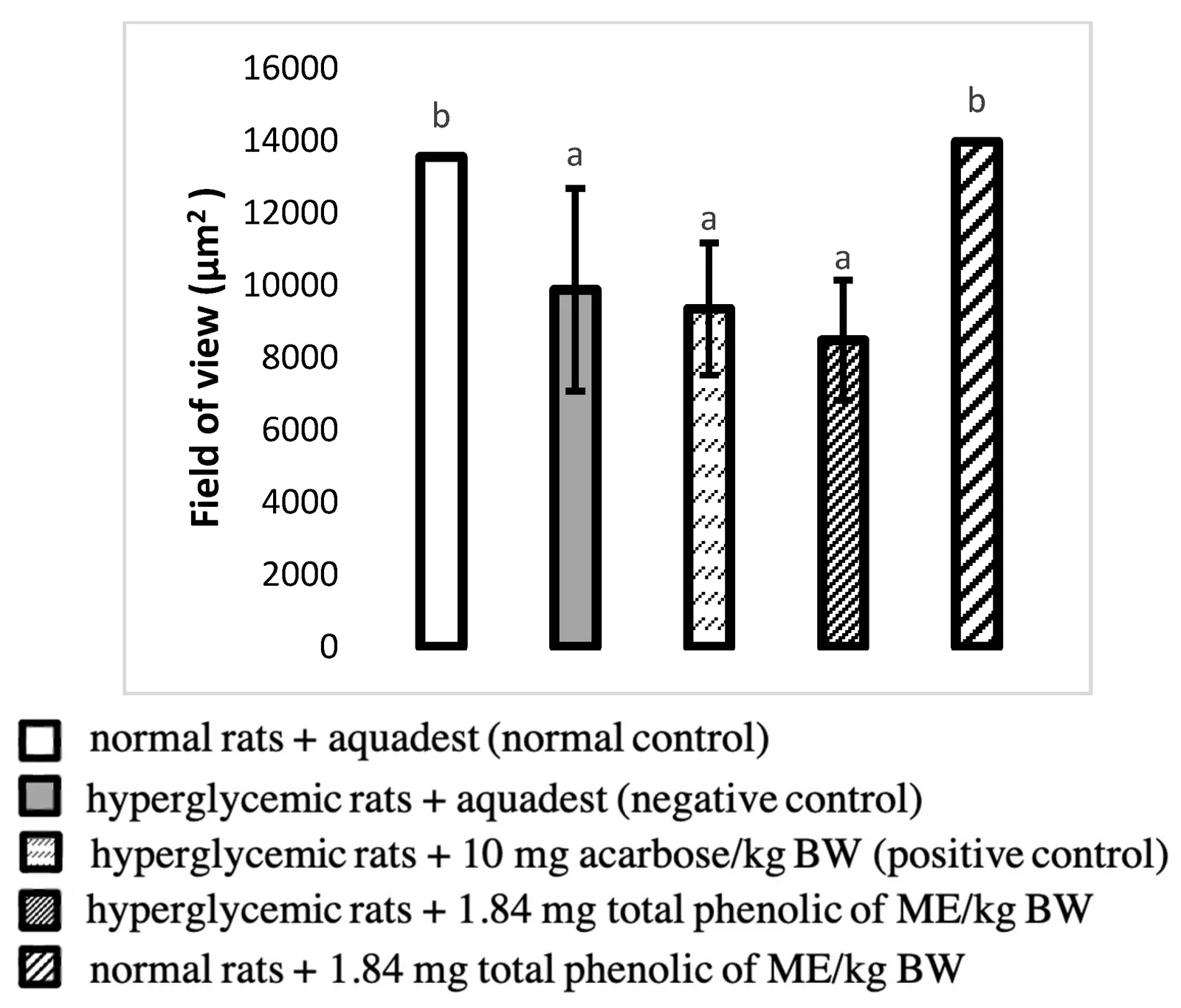
Area of the Islets of Langerhans in pancreatic tissue in normal and hyperglycemic rat groups. Different lowercase letters above bars indicate statistically significant differences (*p* < 0.05).

The area of the Islet of Langerhans was measured (Figure 4) to confirm pancreatic tissue destruction in hyperglycemic rat models and to identify any potential regenerative or protective effects of treatments. All groups of hyperglycemic rats showed significantly smaller areas of the islands compared to normoglycemic rats (*p* < 0.05). At the same time, there was no significant effect of acarbose or ME in alleviating this tissue destruction.

**Figure 5:**
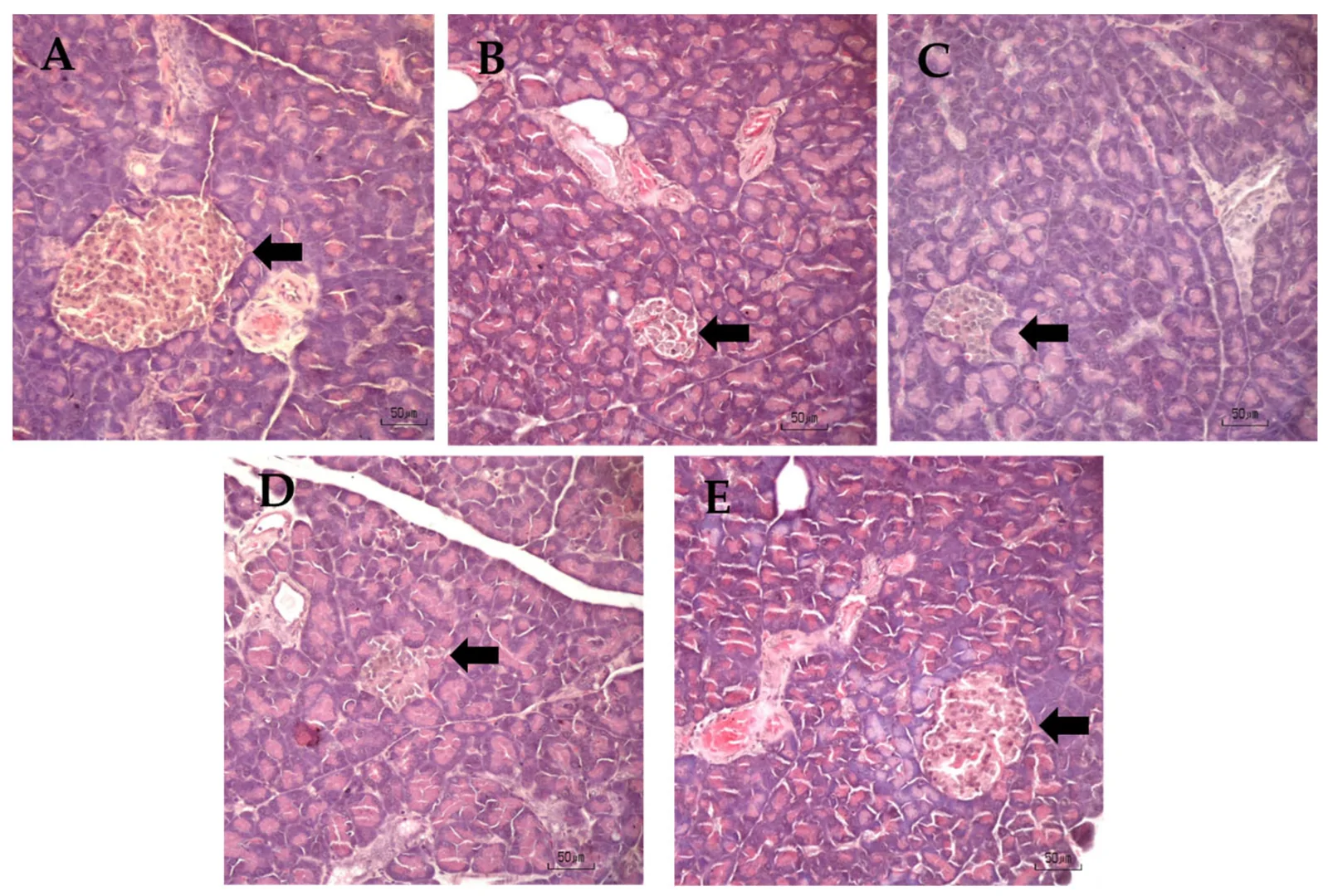
Pancreatic tissue of experimental animals (H&E staining, 20×). The islet of Langerhans (arrow). (**A**) Normal rats receiving distilled water (normal control); (**B**) hyperglycemic rats receiving distilled water (negative control); (**C**) hyperglycemic rats administered acarbose at 10 mg/kg body weight (positive control); (**D**) hyperglycemic rats treated with ME providing 1.84 mg total phenolic compounds/kg body weight; and (**E**) normal rats treated with ME providing 1.84 mg total phenolic compounds/kg body weight.

The tissue profiles of the pancreas of normoglycemic and hyperglycemic animals are shown in Figure 5. The histological analysis of the pancreas revealed distinct morphological differences between the normal control group and the four experimental treatment groups, focusing on both the endocrine and exocrine regions. Hyperglycemic groups displayed a strikingly similar pathological pattern characterized by significantly smaller Islets of Langerhans. This reduction in islet size across the treatment groups was primarily driven by a marked decrease in the overall cell count (i.e., hypocellularity) within the endocrine clusters. There was, however, no indication of lesser destruction with acarbose or ME.

Despite the notable alterations observed in the endocrine tissue, the exocrine portion of the pancreas remained well-preserved across all five experimental groups. The pancreatic acini, secretory cells, and ductal networks showed no signs of inflammation, structural degeneration, or any other morphological abnormalities. Consequently, the findings indicate that the STZ induced a highly selective effect, specifically causing cellular depletion and subsequent atrophy within the Islets of Langerhans while completely sparing the exocrine tissue.

The results of microscopic examination of pancreatic sections conducted by Adeyemi et al. [61] also found that the pancreas of untreated STZ-induced diabetic Wistar rats had micro-anatomical features including degenerative and necrotic changes and shrinkage of the pancreatic islets of Langerhans, β-cell degranulation, pyknotic β-cell nuclei, decreased islet cell density, and severe vacuolization of the islets, as well as a severe reduction in the number of cells in the islets, although the pancreatic acinar epithelium and ductal and connective tissues appeared normal. A significant decrease in the numerical density of the islets (number of islets/pancreatic), islet area, islet diameter, β-cell numerical density (number of β-cells per islet), islet volume, and β-cell volume was also observed in the untreated diabetic rat group compared to the normal rat group through morphometric analysis [61].

The H&E staining method used in this study can easily differentiate between exocrine tissue (pancreatic acini) and endocrine tissue (islets of Langerhans). In fact, in advanced diabetic conditions, H&E can show atrophy or general reduction in the size of the Islets of Langerhans, infiltration of inflammatory cells (especially in type 1 diabetes mellitus (T1DM)), detection of amyloid deposition (insulinoma/fibrosis), and identification of vascular and fatty changes. However, the results of observations of pancreatic organ tissue in this study have not provided strong justification for the effect of ME on target organ repair, especially those related to repairing pancreatic β-cell damage in a group of hyperglycemic mice due to STZ induction [22]. With H&E staining, all endocrine cells, namely β-cells (insulin-producing), α-cells (glucagon), δ-cells (somatostatin), and PP cells, look the same (pale pink cytoplasm with round nuclei) under the microscope, so it cannot be confirmed that the decrease in the mass of the islets of Langerhans is caused by a specific loss of β-cells or other cells. This is H&E’s biggest limitation.

**Figure 6:**
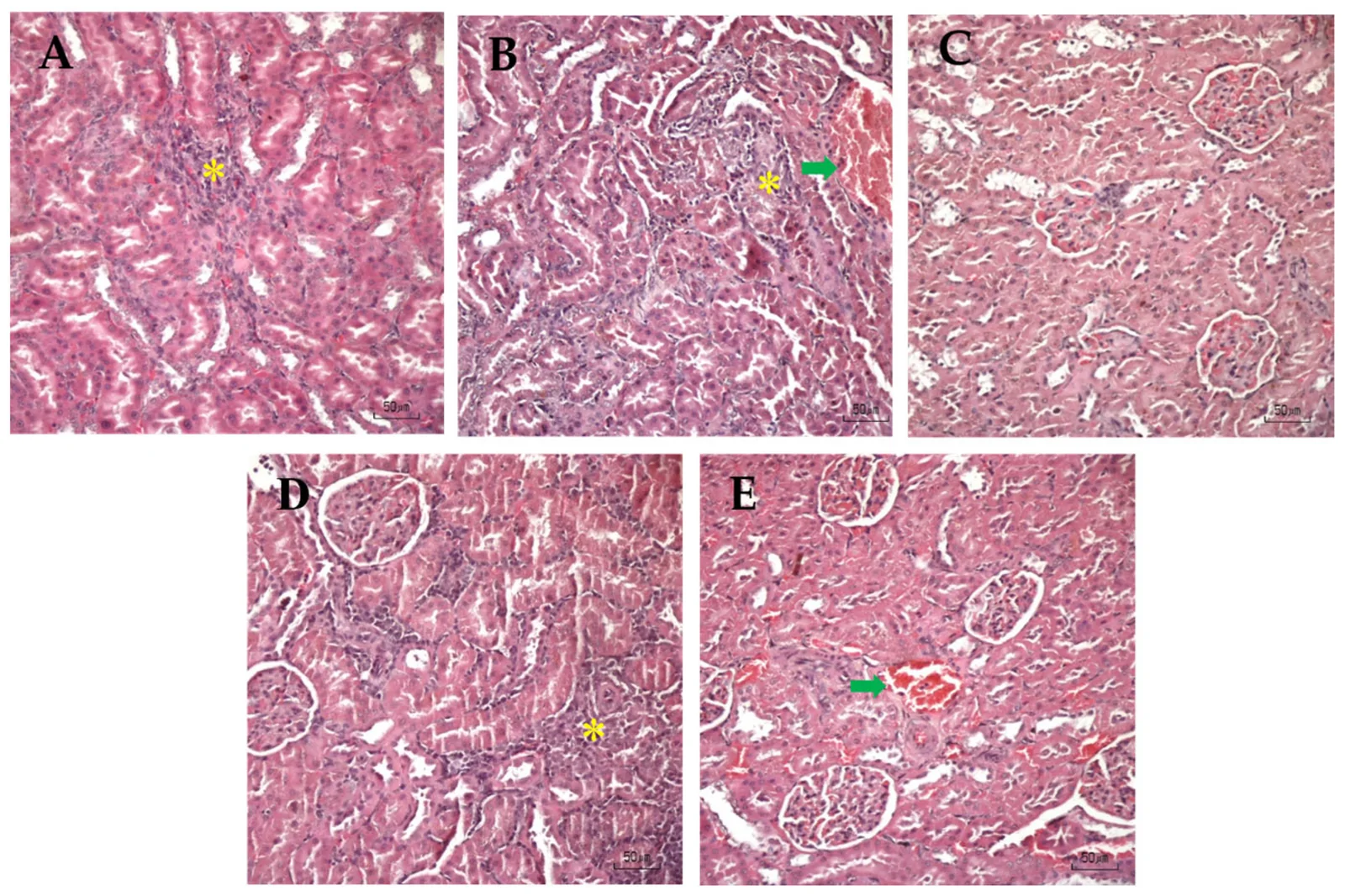
Kidney tissue of experimental animals (H&E staining, 20×). (**A**) Normal rats receiving distilled water (normal control); (**B**) hyperglycemic rats receiving distilled water (negative control); (**C**) hyperglycemic rats administered acarbose at 10 mg/kg body weight (positive control); (**D**) hyperglycemic rats treated with ME providing 1.84 mg total phenolic compounds/kg body weight; and (**E**) normal rats treated with ME providing 1.84 mg total phenolic compounds/kg body weight. The tissue profile was similar in all groups, wherein focal infiltration of mononuclear inflammatory cells within the interstitium (the yellow asterisk) and vascular congestion (arrow) were intermittently found.

**Figure 7:**
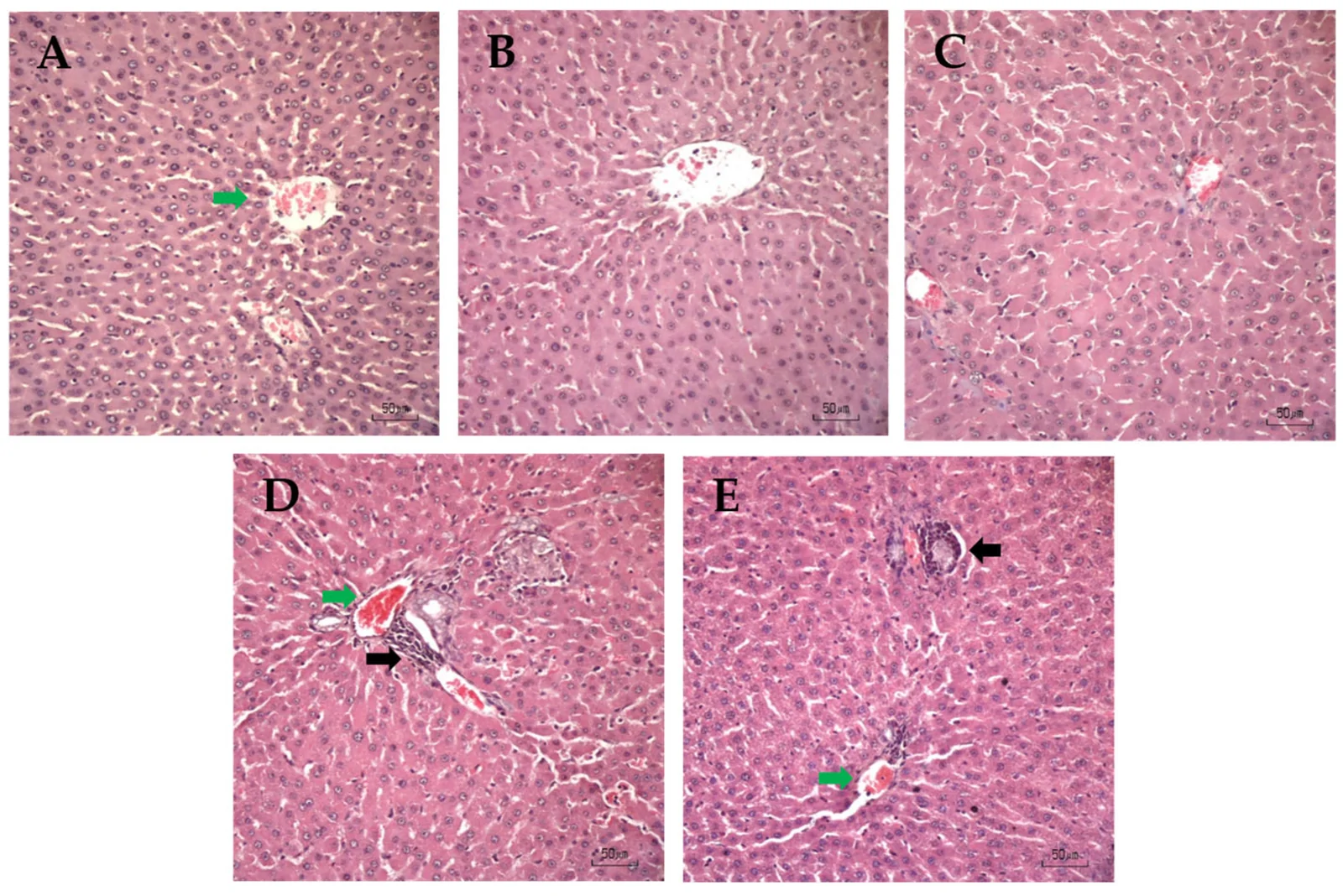
Histopathological description of liver section (H&E staining, 20×). Focal infiltration of mononuclear inflammatory cells (black arrow) and vascular congestion (green arrow) (**D**,**E**); no significant histopathological changes in the normal control group (**A**), negative control group (**B**) and positive control group (**C**). (**A**) Normal rats receiving distilled water (normal control); (**B**) hyperglycemic rats receiving distilled water (negative control); (**C**) hyperglycemic rats administered acarbose at 10 mg/kg body weight (positive control); (**D**) hyperglycemic rats treated with ME providing 1.84 mg total phenolic compounds/kg body weight; and (**E**) normal rats treated with ME providing 1.84 mg total phenolic compounds/kg body weight.

Histopathological profiles of the kidney (Figure 6) and liver (Figure 7) are shown below.

The histopathological examination of the kidney tissue sections revealed a spectrum of normal architecture and pathological variations across different groups. While the normal group demonstrated well-preserved, normal kidney parenchyma with intact glomeruli and unremarkable tubulointerstitial structures, the hyperglycemic groups exhibited signs of focal interstitial nephritis characterized by localized mononuclear inflammatory cell infiltration within the interstitium with vascular lesions featuring kidney vascular congestion, altogether reflecting a progression from normal tissue to localized inflammation and vascular disturbances. No distinctive pattern was found with the extract treatment.

The liver sections exhibited a well-preserved and normal lobular architecture, characterized by hepatocytes arranged in neat radiating cords around an intact central vein, with uniform eosinophilic cytoplasm and clear sinusoidal spaces showing no signs of inflammation or congestion. There were mild signs of focal infiltration of mononuclear inflammatory cells aggregated primarily around the portal triad and vascular structures found in some individuals across all groups. There were no particular signs of hepatic injury related to acarbose or ME treatments.

## 4. Discussion

The aim of the study was to evaluate the potential effects of ME on serum biochemical and histopathological profiles in normoglycemic and STZ-induced hyperglycemic rats. Our results showed that the levels of TG, total cholesterol, and HDL of all groups of hyperglycemic rats were significantly higher than those of the normoglycemic rats, which is consistent with other studies of diabetic rat models [62]. Although the overall lipid profile of rats receiving ME was not significantly different compared to other groups of hyperglycemic rats, the LDL value was significantly lower (by 31.85%) compared to the negative control. This effect was similar to that found with acarbose treatment, although relatively less prominent. Acarbose is an intestinal α-glucosidase inhibitor, where its effect in delaying carbohydrate absorption and limiting postprandial glucose and insulin spikes may contribute to hepatic lipid metabolism, and in turn can influence LDL [63]. A study by Yousefi et al. showed that acarbose can lower serum TG and TC levels. However, no significant effect was found on LDL or HDL levels [64]. It is unlikely that the effect of the ME on lowering the LDL was achieved through a similar mechanism. We have previously reported the antioxidant effect of this extract [36], and further studies are needed to confirm whether the LDL-lowering effect is indeed achieved through modulation of oxidative stress.

Besides classic indicators such as cholesterol and lipoproteins, previous studies have demonstrated associations between plasma levels of free fatty acids (FFA), insulin resistance, and the development of diabetes mellitus [65,66,67]. In recent years, with further research and advancement of testing technologies, the FFA profile has gained widespread attention [68]. It has been shown that in patients with T2DM the total plasma FFA level is elevated [69,70]. The most commonly reported pattern in T2DM patients is an increase in plasma SFAs [71]. Impaired insulin secretion, reduced insulin sensitivity, and impaired glucose tolerance were shown to be closely related to elevated plasma FFA levels, particularly SFAs such as C16:0 and C18:0 [72]. Although the exact mechanisms behind the changes in blood FFA profiles in diabetic patients are not yet fully elucidated, they are suggested to be related to alterations in lipid metabolism [73]. Our study did not involve the identification of the plasma fatty acid profile. Such an analysis would clearly add value to the research presented. Kidney dysfunction is one of the complications associated with diabetes [74]. Elevated blood urea nitrogen (BUN) and creatinine levels, commonly known as azotemia, may be indicators of a lower glomerular filtration rate, which may occur when kidney function is impaired [75,76]. Elevated serum levels of these two biochemical parameters often appear in diabetic nephropathy as persistent hyperglycemia induces oxidative stress in the kidney glomeruli [15]. A study by Draganescu et al. showed that STZ-induced diabetic Wistar rats had elevated serum creatinine, uric acid, and blood urea nitrogen levels compared to the normal group [77]. Here, we found that BUN and creatinine levels in the hyperglycemic rat group were indeed higher than in the normal rat group, indicating impaired kidney function. Interestingly, administration of ME significantly reduced creatinine levels in the hyperglycemic rats (13.78%) compared to the negative control, even to a comparable level as the normoglycemic rats. This positive effect is thought to originate from the phytochemical compounds found in the ME, including eugenol. Nutmeg essential oil, obtained from various parts of the nutmeg plant, including mace, contains a number of phytochemical compounds. Among these is eugenol [47,78,79,80], and Mnafgui et al. have reported the positive effects of eugenol in improving the level of BUN and creatinine, as well as histopathological indices associated with diabetes-induced kidney dysfunction [81].

In this study, our histopathology analysis did not find a positive effect of ME in improving the kidney at the cellular level. The improved serum creatinine level may indicate a possible nephroprotective effect of this extract, which is thought to occur through a mechanism that reduces pro-inflammatory cytokines such as TNF-α and IL-6 in kidney tissue [82]. This ability may originate from the phenolic compounds found in ME, as shown by Al-Rawi et al. that polyphenols in nutmeg prevent thickening of the basement membrane in Bowman’s capsule [83].

The liver plays a role in regulating glycemic homeostasis through glycogenesis, gluconeogenesis, and others [84]. Elevated liver enzyme concentrations (including aminotransferases) are often found with impaired liver function, but it does not always indicate hepatitis, as it also occurs during metabolic syndrome [85]. This elevation of circulating enzymes has been reported to have a high prevalence in diabetic patients (78.4% for T2DM, and 27.3% for T1DM) [86]. A meta-analysis by Kunutsor, Apekey and Walley showed a moderate association between increased risk of T2DM and aminotransferases [87]. Improved aminotransferase enzymes (AST and ALT) in plasma or serum can therefore be a potent indicator of hepatoprotective activity [76,88]. Research by Morita et al. found that AST and ALT activity in the plasma of Wistar rats induced by LPS/D-GalN given nutmeg samples was significantly lower than that of the control diet and 20 other types of spice samples tested in the study. Morita et al. stated that myristicin in nutmeg samples has an extraordinarily potent hepatoprotective activity that significantly suppresses the increase in serum TNF-R concentration and liver DNA fragmentation induced by LPS/D-GalN in rats. The hepatoprotective activity of myristicin is predicted to occur through a mechanism of inhibition of TNF-R release from macrophages [88].

In this study, the hyperglycemic rat group had significantly higher AST and ALT levels than the normoglycemic group. Interestingly, administration of ME to hyperglycemic rats significantly reduced AST and ALT levels to relatively similar levels as the normal control. AST activity of hyperglycemic rats receiving ME was 58.97% lower than the negative control (hyperglycemic without drug/sample) and 74.50% lower than the positive control (hyperglycemic rats given acarbose). Meanwhile, ALT activity in hyperglycemic rats receiving ME was 60.04% lower than the negative control (hyperglycemic without drug/sample) and 68.21% lower than the positive control (hyperglycemic rats given acarbose). These findings indicate a promising hepatoprotective effect of ME, especially in hyperglycemic conditions. This study, however, did not explore the effect of the extract on the inflammatory cytokines. Chronic, low-intensity inflammation is a key contributor to the development and progression of T2DM and may provide an important mechanistic link between diabetes and various comorbidities associated with inflammatory processes. High levels of extracellular glucose increase oxidative stress, which in turn increases ROS production and leads to inflammation, which can be reflected by the level of TNF-α and IL-6 [89]. Future studies are needed to confirm the effect of ME on modulating inflammatory cytokine levels in hyperglycemic rats.

Albumin is a protein that functions to maintain plasma osmotic pressure, a means of transporting bilirubin, fatty acids and drugs. The absolute level of this protein is influenced by age, nutrition, and disease. Plasma albumin concentration is related to metabolic disorders such as diabetes mellitus and metabolic syndrome. In theory, patients with uncontrolled diabetes have low plasma albumin levels [90]. Albumin deficiency in the blood, alongside high albumin concentration found in the urine, is often caused by kidney damage due to diabetes. Chronic hyperglycemia slowly damages the kidney’s filtering membrane, the Bowman’s capsule. This will result in albuminuria, a condition in which high levels of albumin are found in the urine, which is often referred to as a sign of kidney leakage [91]. Research by Purba et al. [91] found that as many as 75% of T2DM patients have lower-than-normal circulating albumin levels. Notably, albumin is synthesized by the liver. Therefore, a low level of albumin in the blood may also be an indication of a chronic liver problem. In this study, albumin levels in all hyperglycemic groups were 39.17% lower than those of normal controls. However, we did not find any effect of ME in improving the circulating albumin level.

It should be acknowledged that severe weight loss is a common characteristic of diabetes mellitus, resulting from the loss or degeneration of structural proteins. Our previous article [38] reported that rats in all hyperglycemic groups experienced weight loss at various time points, whereas similar weight loss did not occur in the normal control group. By the fourth week (the end of the study period), weight loss (17.48–18.93%) was recorded in all hyperglycemic groups. In the first week, weight loss reached 12.04% and 10.67% in the negative and positive control groups, respectively, whereas it was only 5.44% in the hyperglycemic group receiving the ME intervention. By the second week, weight loss was 16.67% and 18.52% in the negative and positive control groups, respectively, compared to 11.46% in the hyperglycemic group receiving the ME intervention. The comparatively lower weekly reduction in body weight observed in hyperglycemic rats treated with ME (13.33 g/week), relative to those in the negative control (19.83 g/week) and positive control (20.83 g/week) groups, may indicate a protective or restorative effect of ME against excessive activation of gluconeogenesis and glycogenolysis associated with hyperglycemia [38].

Our previous study [38] demonstrated that acute administration of ME at a dose equivalent to 1.84 mg total phenolic compounds/kg BW attenuated the postprandial increase in blood glucose during both oral starch and glucose tolerance tests, as evidenced by a lower area under the curve (AUC) than that observed in the negative control group. Furthermore, following 28 days of ME treatment, streptozotocin-induced hyperglycemic rats exhibited reduced fasting blood glucose and HbA1c levels compared with the negative control group, with the observed reductions also exceeding those achieved in the positive control group. These findings provided the first indication that ME has antihyperglycemic potential in vivo and could be considered as a functional food ingredient.

## 5. Conclusions

Overall, this study confirmed that supplementation of ME (equivalent to 1.84 mg total phenolics/kg BW) was effective in mitigating some serum biochemical dysregulation triggered by hyperglycemia. Significant reductions in LDL (31.85%), creatinine (13.78%), and transaminase enzymes AST (58.97%) and ALT (60.04%) indicated a possible hepatoprotective and nephroprotective capacity. These results validate the potential of ME as a candidate supportive agent for the management of metabolic complications and a functional ingredient for the development of functional food products.

The novelty of the present study resides in the first comprehensive assessment of the metabolic, biochemical, and histopathological effects of an aqueous *Myristica fragrans* mace extract in hyperglycemia, extending previous research based on organic solvent extracts and highlighting the translational potential of a safe, food-compatible extraction strategy for functional food and nutraceutical applications. Further extract characterization would be important to provide insight into the specific mace compounds contributing to ME’s antihyperglycemic activity.

## Data Availability

The original contributions presented in this study are included in the article. Further inquiries can be directed to the corresponding authors.

## Abbreviations

The following abbreviations are used in this manuscript:

AGEs	advanced glycation end products
ALT	alanine aminotransferase
ANOVA	analysis of variance
AST	aspartate aminotransferase
BUN	blood urea nitrogen
BW	body weight
D-GalN	D-galactosamine
DNA	deoxyribonucleic acid
GLUT	glucose transporter
H&E	hematoxylin and eosin
HbA1c	glycosylated hemoglobin
HDL	high-density lipoprotein
IL-6	Interleukin-6
LDL	low-density lipoprotein
LPS	lipopolysaccharide
ME	mace water extract from *Myristica fragrans* Houtt
MASLD	metabolic dysfunction-associated steatotic liver disease
PP cells	pancreatic polypeptide cells, also known as F or gamma cells
ROS	reactive oxygen species
STZ	streptozotocin
T1DM	type 1 diabetes mellitus
T2DM	type 2 diabetes mellitus
TC	total cholesterol
TG	triglycerides
TNF-R	Tumor Necrosis Factor-receptor
TNF-α	Tumor Necrosis Factor-alpha
